# Supplementary material for: A Conceptual Model Depicting How Children Are Affected by Parental Cancer: A Constructivist Grounded Theory Approach
Source: Children (Basel). 2023 Sep 4;10(9):1507. doi: 10.3390/children10091507 (PMC10530185; doi:10.3390/children10091507)
Supplement: Supplementary file 1 [file children-10-01507-s001.zip › children-2508921-supplementary.pdf]

Supplementary file S1. Topic guide for qualitative interviews

Health Professionals

Number	Question	Prompts
1	Tell me about your contact and involvement with patients' children?	*Experiences*
2	What do children come in for? What do they talk about with you?	*What is life like for these children?*
		What is going on for them?
3	How do you feel patients' children are affected by their parents' cancer?	
4	What do you think would help patients and their children?	
5	What are your concerns for the wellbeing of patients' children?	
6	What supports do you feel children need to adjust to their parent's cancer diagnosis and treatment?	*Are these supports available*
		What supports are available?
7	Are patients' children supported in clinical practice?	
8	How might staff be assisted in providing support for children?	*How would you suggest these children could be better supported and assisted?*
9	Is there anything else you would like to say?	

Parents

Number	Question	Prompts
1	Can you tell me a bit about who is in your family?	*Such as who is in your family? Do you have any pets?*
2	What activities do you and your family enjoy doing together?	
3	Has any of this changed since your/your partner's diagnosis?	
4	Tell me about your cancer diagnosis	
5	What are the key challenges you have faced since your/your partner's diagnosis?	
6	Do you feel okay talking to your children about your/your partner's cancer and any changes?	*[If not]* Okay, why is that?<br>*How have you talked to your children?*
7	How do you think your child/ren has/have been affected by your/your partner's cancer? How do you	

	feel your child/ren has/have coped with your/your partner's cancer diagnosis?	
8	Have you noticed any other changes in your child's/children's behavior?	
9	What, if any, worries or concerns do you have for your child/ren and their coping with your/your partner's cancer?	
10	Do you feel confident and comfortable with supporting and assisting your child/ren to cope with your/your partner's cancer, and any problems or issues that might arise?	*What support have you had?*
11	Is there anything that might make you feel more comfortable to do this?	
12	What are the main challenges you face with supporting your child(ren)?	*Is there anything else you would like to say?*

Children

Number	Question	Prompts
1	Can you tell me about your family?	Such as who is in your family? Do you have any pets?
2	What are the fun things your family enjoy doing together?	Have any of these things changed lately?
3	Is there anything that you worry about?	
4	I was hoping you could tell me a little bit about your [mum/dad]. Has [mum/dad] been sick lately?	
5	What do you call [mum's/dad's] sick/sickness?	
6	Tell me what you know about [mum's/dad's] sickness?	
7	If you have a question about [mum's/dad's] sickness, who do you ask or what do you do?	
8	Is mum and dad OK talking to you about [mum/dad] not being well?	*[If yes]* Tell me some of the things you talk about with mum and dad?

		[If no] Would you like to be able to talk to mum and dad about this more?
9	Are there more things you want to know about [mum's/dad's] sickness?	*[If yes]* Tell me what sort of things?
	What are some things you do to help you feel better about [mum/dad] not being well?	
	Has life been different since [mum/dad] found out [he/she] was not well?	*[If yes]* Tell me how it has been different?
	Are things still the same with your friends, or have they changed?	*[If they have changed]* Tell me how they have changed?
	Are things still the same at school, or have they changed?	*[If they have changed]* Tell me how they have changed?
	Is there someone at school you prefer to talk to about [mum/dad] not being well?	*[If yes]* Tell me who this person is?
	What makes you feel the happiest lately?	[Prompt] Activities? Things? Items? People?
	And, what makes you feel unhappy or sad lately?	[Prompt] Activities? Things? Items? People?
	If I asked you to do a special activity with mum or dad, and it could be any kind of activity, what would that special activity be?	
	If you had a friend that found out their mum or dad was not well in a similar way to your [mum/dad], what would you do to help that friend?	
	If you had 3 wishes, what would those wishes be?	
